# Detection and genetic diversity of parechoviruses in children with acute flaccid paralysis in Cameroon

**DOI:** 10.1371/journal.pone.0301771

**Published:** 2024-05-29

**Authors:** Daniel Kamga Njile, Emmanuel Akongnwi Mugyia, Marie Claire Endegue-Zanga, Jude Anfumbom Kfutwah, Marlise Dontsop Djoumetio, Boyomo Onana, Ousmane Madiagne Diop, Richard Njouom, Serge Alain Sadeuh-Mba

**Affiliations:** 1 Department of Virology, Centre Pasteur of Cameroon, Yaoundé, Cameroon; 2 Faculty of Sciences, Department of Microbiology, University of Yaoundé 1, Yaounde, Cameroon; 3 Faculty of Science, Department of Microbiology and Parasitology, University of Buea, Buea, Cameroon; 4 World Health Organization, Regional Office, Libreville, Gabon; 5 World Health Organization, Country Office, Yaoundé, Cameroon; 6 Global Polio Eradication Initiative (GPEI), World Health Organization, Geneva, Switzerland; Children’s National Hospital, George Washington University, UNITED STATES

## Abstract

Human Parechoviruses (HPeVs) have rarely been considered in the virological investigation of Acute Flacid Paralysis (AFP) cases in Africa, where enteric infections are very common. This study investigated the prevalence and genetic diversity of HPeV in 200 children aged ≤ 15 years with AFP in Cameroon from 2018 to 2019. HPeVs were detected in their faecal RNA using 5’-untranslated real-time RT-PCR. Detected HPeVs were typed by phylogenetic comparison with homologous sequences from homotypic reference strains. Overall, HPeV RNA was detected in 11.0% (22/200) of the 200 stool samples tested. Twelve HPeVs were successfully sequenced and reliably assigned to HPeV-A1, A4, A5, A10, A14, A15, A17 and A18 genotypes. Phylogenetic analyses revealed a high genetic variability among the studied HPeVs, as well as between the studied HPeVs and their previously reported counterparts from Cameroon in 2014. These findings suggest that different HPeV genotypes co-circulate in Cameroon without documented epidemics.

## Introduction

Human Parechoviruses (HPeVs) were first discovered 50 years ago during a winter epidemic of diarrhoea in young children [1]. HPeVs are small, non-enveloped viruses with an icosahedral capsid of approximately 28 nm in diameter, consisting of 60 capsomers [2]. Each capsomere is composed of 3 distinct polypeptide chains of viral protein (VP): VP0, VP1 and VP3 [3]. The HPeV genome consists of a single-stranded positive-sense RNA of approximately 7300 nucleotides. It consists of a single reading frame that is translated into a polyprotein encoding 3 structural proteins and 7 non-structural proteins [2].

Previously identified by serotyping as Echovirus types 22 and 23 in 1956, it wasn’t until 1999 that HPeV were assigned in a separate genus based on their growth characteristics, serological and biological properties as well as genome analysis [4,5]. HPeVs belong to the family *Picornaviridae*, genus *Parechovirus*. The genus *Parechovirus* contains 6 recognized virus species: *Parechovirus A*, *B*, *C*, *D*, *E* and *F*. HPeVs which only infect humans, belong to the species Parechovirus A and consist of 19 types, HPeV-1 to -19. In contrast to HPeV B to F, which infect animals, HPeVs A are the most described aetiological agents of diseases in children [6]. In fact, the 19 currently recognized genotypes of HPeV-A can cause mild respiratory or gastrointestinal symptoms as well as severe disease, such as HPeV A-3 induced encephalopathy, meningitidis and acute flaccid paralysis (AFP) [2,7]. Several central nervous system epidemics due to HPeV have been reported worldwide. For instance, an outbreak was recently reported among infants aged between 5 days and 3 months hospitalized in Tennessee, United Sates of America (USA), with HPeV-associated meningoencephalitis in 2022 [6]. Since 2017, an increase in central nervous system infections due to HPeV in young infants led to the proposition to include molecular diagnostic techniques for early detection of HPeV in the standard practice for investigation of sepsis-like illnesses and CNS infections in that age group in USA [7].

AFP is defined as acute or sudden onset of weakness or paralysis of a limb, characterized as flaccid, in a child < 15 years of age [8]. AFP surveillance consists of the detection and virological investigation of flaccid paralysis in children under 15 years of age or any other suspected case of poliomyelitis case in a person of any age [9]. It has been adopted globally as an essential strategy for monitoring the progress of the poliomyelitis eradication initiative [10]. Hence, AFP surveillance remains the “gold standard” for identifying high-risk areas and populations, assessing the poliomyelitis status of a country, identifying the need for immunization campaigns, and certifying the absence of wild poliovirus circulation in countries that have stopped reporting poliomyelitis cases [11]. Historically known to be caused by enteroviruses since ancient Egypt, AFP is now recognized to be associated with other infectious and non-infectious etiologies such as traumatic, toxic and metabolic causes [12]. Besides enteroviruses, other enteric viruses colonize the gastrointestinal tract, where they can multiply and subsequently migrate to secondary organs, including the central nervous system (CNS), where they can induce AFP [2].

Although the involvement of enteric viruses in neurological diseases can hardly be excluded, studies from Italy, Nigeria and Gabon reported HPeVs, rotavirus and astroviruses among AFP cases [13–16]. A study conducted in Iran in 2019 showed that several types of enteric viruses, including HPeV A-1, were associated with AFP [17]. In particular, HPeV A-3 has been documented as an aetiological agent of AFP [7].

With the development of molecular techniques, many enterovirus diagnostic laboratories have replaced cell culture with reverse transcription polymerase chain reaction (RT-PCR) based on the highly conserved 5′ untranslated region (5′UTR) to detect enterovirus in cerebrospinal fluid (CSF), swab and stool specimens. However, as cell cultures, 5′UTR region-based RT-PCR, which is optimized for enterovirus detection, does not detect HPeV as it is in cell culture. Moreover, RT-PCR assays for HPeV are not routinely used to detect HPeV in clinical samples. This could lead to misdiagnosis or underestimation of HPeV infection in epidemiological settings such as Cameroon, where extensive enterovirus circulation has been documented [18]. Real-time RT-PCR (rRT-PCR) assays targeting the 5’-UTR remain the most sensitive and specific molecular method for diagnosis of HPeV infection using specific primers/probes that do not cross-react with other viruses, including enteroviruses [19]. To ensure the specificity of HPeV detection, several authors have optimized existing protocols for the detection of HPeV in diverse pathological specimens [20–22]. After the first-line 5’-UTR-based diagnosis of HPeV infection, molecular typing of field HPeV is usually performed by genetic comparison of their viral protein 1 (VP1) derived nucleotide sequence with that of reference strains retrievable from databases. Nucleotide sequences of the VP1 of homotypic isolates display ≥77% similarity to each other [23].

One of the WHO-specified AFP surveillance indicators requires that at least 10% of stool samples from AFP cases submitted to the laboratory should be positive for non-polio enteroviruses in Human rhabdomyosarcoma (RD) cell cultures [24]. This is rarely achieved in African settings, including Cameroon, where these unexpected results have been interpreted as a consequence of poor storage of specimens at the operational level, as well as failure to maintain the cold chain during specimen transport [9,25]. The low frequency of enterovirus isolation from AFP cases in cell culture may also be the result of infection with other enteric viruses with secondary neurological tropism, such as HPeVs. The prevalence of HPeVs has been shown to be high in diverse epidemiological settings worldwide. An HPeV prevalence of 25% was reported in Asia, with HPeV-A1 and HPeV-A3 being the most frequent genotypes [26]. In Europe and the USA, the prevalence of 1 to 7% has been reported, with a relatively higher frequency of HPeV‐A1, A3, A4, A2, while genotypes HPeV‐A7 to 19 have been rarely detected [27–29]. Extensive HPeV circulation has been documented in some African countries such as Ethiopia, Egypt and Malawi with HPeV detection rates of 10.9%, 19% and 57% respectively [30–32]. Despite the limited number of studies published in Africa, all known HPeV genotypes have been described in African studies [30,31,33,34].

In Cameroon, HPeVs detection have been rarely documented. Only one previous study performed detection and molecular typing of two HPeV A-1 and a HPeV A-16 isolates from gastroenteritis and diarrhoea cases in a very limited area of the Centre region of Cameroon [35]. There are no data on HPeV circulation in children with AFP in Cameroon.

### Objectives

The aim of this study was to determine the occurrence and genetic variability of HPeV in AFP cases enrolled in the national poliomyelitis surveillance programme in Cameroon.

## Materials and methods

### Study design

This was a retrospective cross-sectional study carried out on a biological collection of 200 stool samples collected from 200 AFP cases from January 2018 to December 2019. AFP cases were children ≤ 15 years with AFP throughout the national territory of Cameroon. Their stool samples were collected after the medical consultation and sent in a reverse cold-chain (2–8°C) to the Centre Pasteur du Cameroun (CPC), a WHO-accredited Intercountry reference laboratory for poliomyelitis. Cameroon has a wide climatic and agro-ecological diversity. The northern regions are dominated by savannah and steppe, the Centre, South and Est regions are marked by dense rainforests; the West and Northwest regions are made up of highland mountain forest; and the South West and Littoral regions are coastal areas bordering the Atlantic Ocean. The northern part of the country has a short rainy season occurring from April to July, while the equatorial climate in the rest of the country alternates between two rainy and dry seasons, with precipitations in March-June and September-November [36].

### Sample selection

Upon arrival of stool samples at the CPC, viral isolation of polioviruses and non-polio enteroviruses in cell culture was performed according to WHO-specified protocols [37–39]. A total of 200 samples (from 200 AFP children) were randomly selected from 1552 samples previously found negative for enterovirus isolation on three different cell lines: human rhabdomyosarcoma (RD), human larynx epidermoid carcinoma (HEp-2c), and murine L20B (a derivative of murine L cells expressing the human poliovirus receptor). Selection was also based on the month of disease onset and the region of origin of the case.

### Nucleic acid extraction and detection of HPeV RNA by real-time RT-PCR

Total fecal RNA was extracted from 20% (weight/volume) of clarified stool suspensions using the Quick-RNA Viral Kit (ZYMO Research) according to the manufacturer’s instructions [40]. The extracted RNA was eluted in 50 μL RNAse/DNAse free water and stored at -80°C until tested for HPeV RNA by rRT-PCR. Molecular detection of HPeV RNA was performed on Biorad CFX CFX96 (Real-Time PCR Detection System—Gene) as previously described [41] using the primer and probe set described by Brouwer in 2019 [32]. Overall, all stool samples with sigmoidal amplification signals and a cycle threshold (CT) < 35 were considered positive.

### RT-nested PCR, sequencing, and phylogenetic analyses of HPeVs

All RNA samples with CT< 37 on rRT-PCR were further subjected to RT-nested PCR targeting a 520 nucleotide portion of the VP1 coding gene as previously described [42]. Five microliters of PCR products were analysed on Gelgreen® (Invitrogen, Carlsbad, CA, USA) stained agarose gel and visualized under an ultraviolet transilluminator.

Resulting amplicons were purified using the QIAquick PCR pufication kit (Qiagen, Courtaboeuf, France) and subjected to direct sequencing of both strands using the BigDye terminator v3.1 kit (Applied Biosystems, Foster City, CA, USA) and nested PCR primers on an ABI Prism 3140 automated sequencer. Sequence contigs, consensus sequences and multiple sequence alignments were generated using CLC Main Workbench 21.0.3 software (Qiagen, France). Newly determined sequences were submitted to the GenBank database under the nucleotide sequence accession numbers OR078518 and OR078529. Maximum likelihood phylogenetic analysis was performed with MEGA version 7.0 software using the best fit GTR+G+I+4 model. The reliability of individual tree topology was estimated using 1,000 bootstrap pseudo-replicates.

### Data analysis

Microsoft Excel (Microsoft, Washington, DC, USA) was used for statistical analysis. Anonymized epidemiological data were combined to laboratory results, cross-checked, and cleaned for erroneous entries before analyses. Epidemiological analyses were performed focusing on prevalence and seasonality. Prevalence rates were compared using a chi-square (χ^2^) test to determine the P-value. When this probability was ≤ 5%, the difference was statistically significant.

### Ethics statement

This retrospective study was reviewed and approved by the Regional Ethics Committee of Research for Human Health (RECRHH) of the Centre region of Cameroon, under the number CE N° 2374/CRERSHC/2021. Informed consent was waived by the ethics committee from the parents or guardians of the study participants, who were initially enrolled by the national system for poliomyelitis surveillance and control in Cameroon. Authors had no access to information that could identify individual participants during or after data collection and stool samples selection from the biological collection of Centre Pasteur du Cameroon. Anonymous participants data were retrieved from the poliomyelitis surveillance database from July 1 to December 31, 2021.

## Results

### Sociodemographic characteristics of the studied AFP cases

Studied AFP cases were randomly selected among 1552 eligible cases by random selection after stratification by region of origin and month of disease onset. Overall, 200 stool samples from 200 AFP cases ≤ 15 years of age were selected from January 2018 to December 2019. Among the selected patients, 146 children had ages ranging from 3 months to 15 years with a mean of 4.64 ± 1.16 years (Table 1).

**Table 1:** Demographic, epidemiological, and virological characteristics of the study population of acute flaccid paralysis children originating from Cameroon from 2018 to 2019.

Parameters	Total	Negative (%)	Positive (%)	P- value ^&^
	200	178 (89)	22 (11)	
Gender				
Male	125 (62.5)	106 (59.6)	19 (86.4)	
Female	75 (37.5)	72 (40. 4)	3 (29.6)	0.06
Age				
Median [IQR] ^*^	4.64 [3.5–5.8]	4.67 [4.25–5.1]	4.46 [3.42–5.5]	
Age group (year)				
[0–2[	26 (17.8)	23 (17.9)	3 (16. 6)	
[2–5[	65 (44.5)	57 (44.5)	8 (44. 4)	0.026
[5–15]	55 (37.7)	48 (37.5)	7 (39)	
Missing ^#^	54 (27)	50 (28)	4 (18.2)	
Year of disease onset				
2018	109	97 (54.5)	12 (54.5)	
2019	91	81 (45.5)	10 (45.5)	0.028

^*^ Confidence interval at 5% alpha risk of being wrong

^#^ Children with missing age information are accounted as ’’Missing’’

^&^ P values ≤ 0.05 were considered statistically significant for comparisons of the respective variables.

The proportion of age groups varied from < 2 years (26; 17.8%); to 2 to < 5 years (65; 44.5%); and 5–15 years (55; 37.7%). A total of 125 (62.5%) patients were male while 75 (37.5%) were female.

### HPeV prevalence in children with AFP

The baseline characteristics of sex, age, and the prevalence of HPeV detection were statistically comparable during the 2-year study period (Table 1). Overall, 11% (22/200) of the samples tested were positive for HPeV RNA (Table 1). The most affected age group was 2 to < 5 years, thus suggesting that children and infants may be at higher risk of HPeV infection [27,28,43]. Age data were missing for 54 AFP cases and were therefore excluded from the analysis of the association between age and HPeV detection. There was a significant association between the prevalence of HPeV RNA detection and both age and year of detection (P < 0.05). The mean age of HPeV-positive children was 4.46 years (Table 1). The proportion of HPeV-positive children was highest in children aged 2 to < 5 years (8/22, 44.4%) and lower in the other age groups.

### Temporal distribution of HPeV detected in patients with AFP

**Fig 1:**
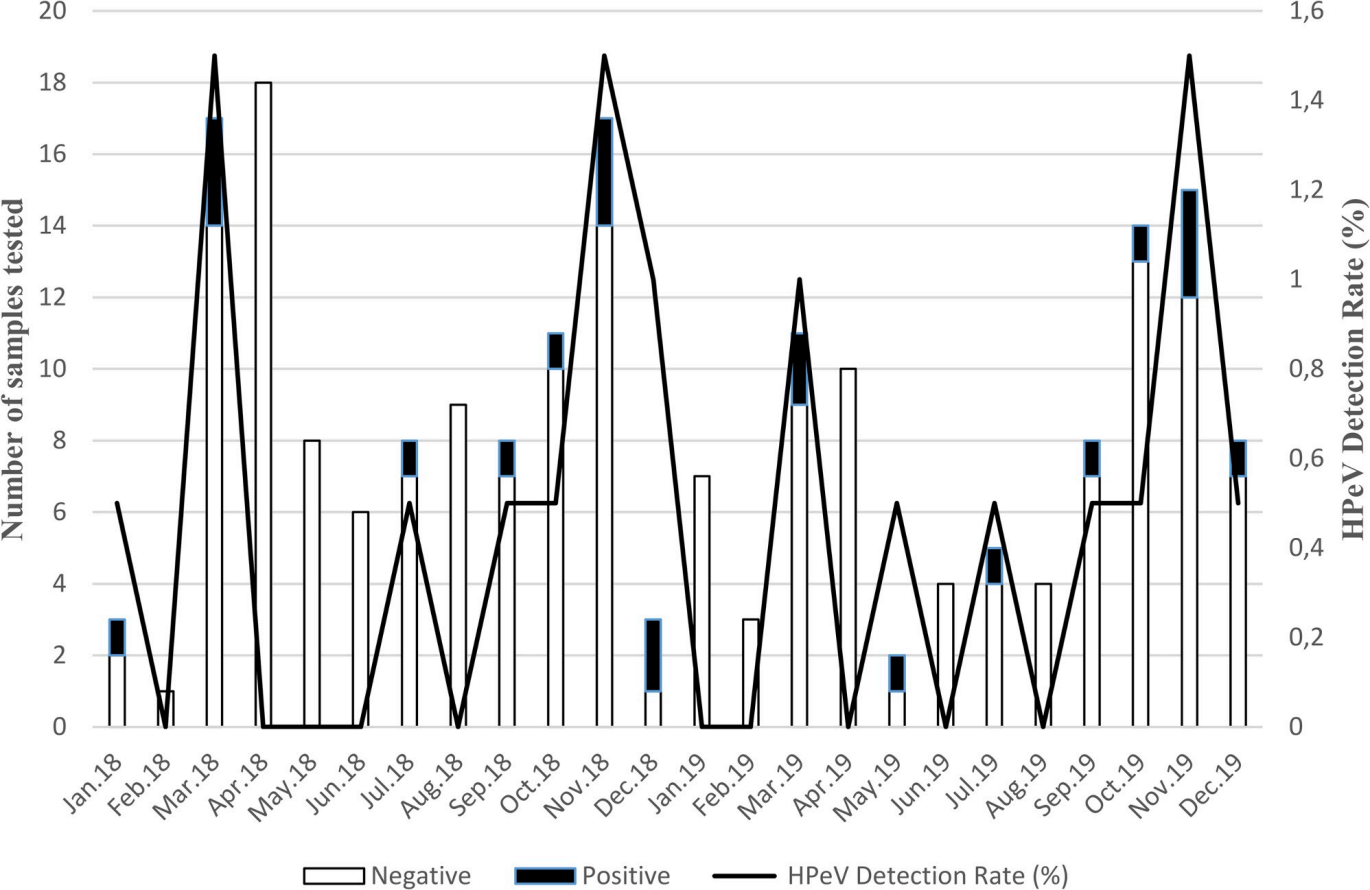
Monthly distribution of human parechoviruses in children with acute flaccid paralysis in Cameroon from January 2018 to December 2019. The primary left y-axis and bars describe the number of stool specimens tested whereas the secondary right y-axis and lines describe the monthly HPeV detection rate.

Monthly distribution of HPeV detected in AFP cases is summarized in Fig 1.

HPeVs were shown to have circulated in Cameroon throughout the study period regardless of their origin and month of collection. HPeV had circulated sporadically during most of the 24 months of the study period. Peak detections were observed during the months of March and November. These findings suggest an intense circulation of HPeVs during the rainy season. Although the seasonal pattern was not strongly evident in this study, the temporal distribution of HPeV detection prevalence was somewhat similar between the 2018 and 2019 study years. Interestingly, both HPeV detection and AFP occurrence were significantly more prevalent during rainy seasons (P value <0.05).

### Genotype assignment and phylogenetic relationships of the detected HPeVs

**Fig 2:**
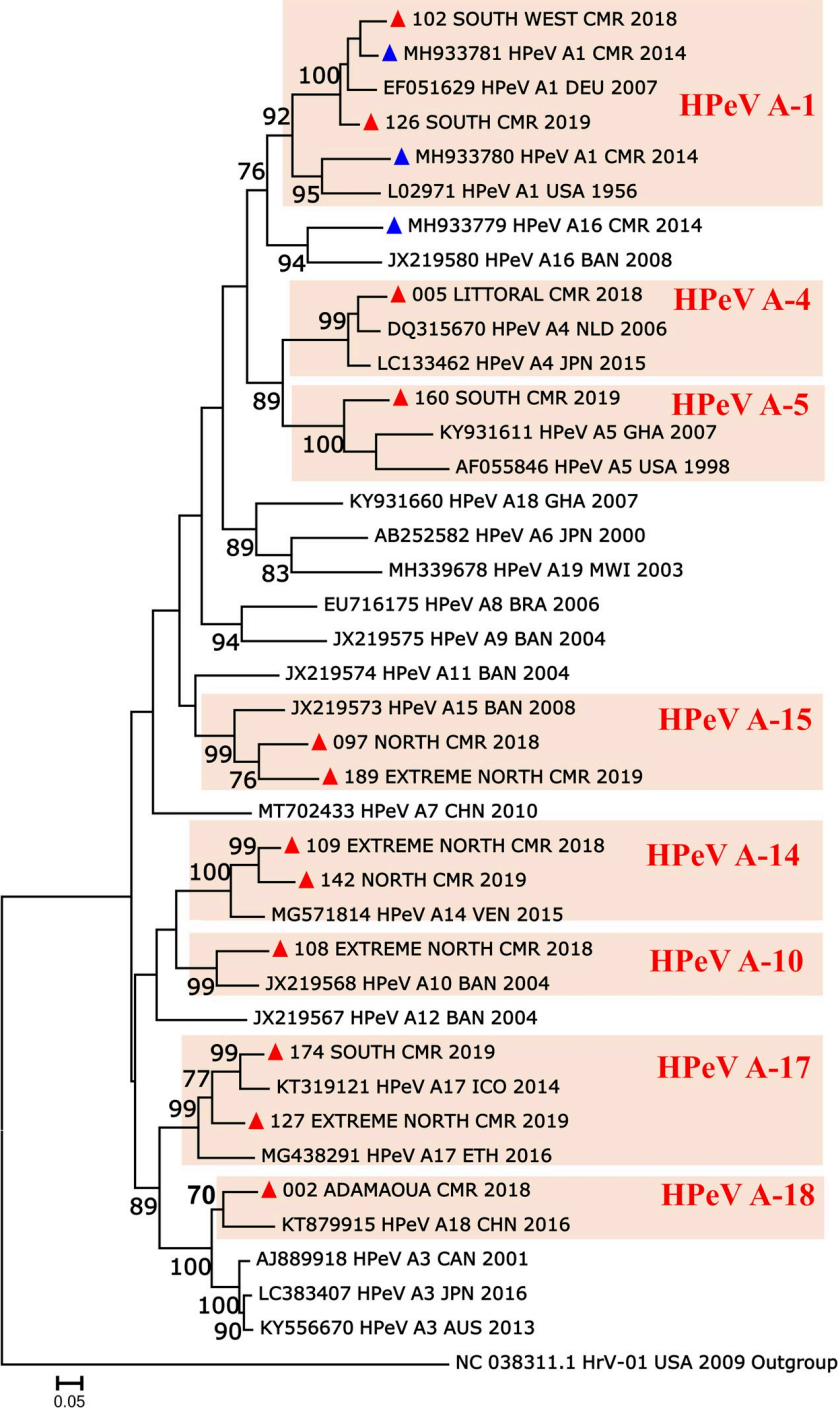
Maximum-likelihood phylogenetic tree of the partial VP1 coding gene depicting the phylogenetic relationships of the HPeVs from Cameroon. The phylogenetic tree was estimated from a 480 nucleotide (nt) sequence alignment using the maximum-likelihood (ML) method with the best-fit nucleotide substitution model (GTR+G+I+4) determined by Smart Model Selection implemented in the PhyML software. Newly sequenced HPeV are indicated by a red triangle (▲) while previously reported sequences from Cameroon are indicated by a blue triangle (▲). Sequences originating from Cameroon are labelled according to the country with the respective laboratory serial number, region of origin, country code CMR and year of sampling. Country names have been abbreviated according to ISO 3166–1 standard: AUS, Autralia; BAN, Bangladesh; BRA, Brazil; CAN, Canada, DEU, Germany; GHA, Ghana; JPN, Japan; CHN, China; ETH, Ethiopia; MWI, Malawi; ICO, Ivory Coast and USA, United States of America. The GenBank accession number, genotype, location, and year of sampling of reference sequences are indicated on the tree. The human rhinovirus A1 (Acc. N° NC_038311) was used as an outgroup for the orientation of the tree. For clarity, most bootstrap values below 70% have been omitted. Scale bars indicate the nucleotide distance as substitutions per site.

Overall, 22 HPeV positive samples with CTs < 35 were available for molecular typing in this study. Following nested RT-PCR targeting the VP1 region, 13 of the 22 positive specimens tested were successfully amplified and sequenced. Usable sequences were obtained from 12 of the 13 amplified samples, while the remaining amplicon showed an unexploitable electrophoregrams with superimposed peaks. Basic Local Alignment Search Tool (BLAST) performed on the NCBI website showed nucleotide sequence identity ≥ 92% with homologous sequences of homotypic sequences from GenBank. Altogether, pairwise sequence comparison and phylogenetic analyses assigned the newly sequenced HPeVs to genotypes HPeV A-1, A-14, A-15 and A-17 with the same frequency of 16.7% (2/12), followed by the less frequent HPeV A-4, A-5, A-10, A-18 with the same frequency of 8.3% (1/12) respectively (Fig 2).

As expected, all HPeV studied isolates fell into different phylogenetic groups defined by their homotypic reference isolates (Fig 2). Apart from the two studied HPeV A-1 sequences, which were strikingly close to each other and to the MH933780 and MH933781 sequences detected in Cameroon in 2014, other studied sequences were somewhat genetically distinct. The identified genotypes segregated into 7 of the 19 currently recognized HPeV genotypes: A-4, A-5, A-10, A-14, A-15, A-17, and A-18. They were close to sequences documented in other countries such as the Netherland, Ghana, Bangladesh, Venezuela, Ivory Coast and China between 2004 and 2015. These phylogenetic results suggest that the high prevalence of HPeV detection was associated with the co-circulation of a wide range of different genetic variants of HPeV, rather than a monophyletic lineage. This is consistent with the fact that the studied HPeVs were not recovered from a specific disease outbreak.

## Discussion

This study is the first to report the prevalence and genetic diversity of HPeV strains detected in children with AFP in Cameroon. We found an overall HPeV prevalence of 11.0% throughout the study period between January 2018 and December 2019. This study showed a high rate and genetic diversity of co-circulating HPeV in Cameroon. This could be hypothetically explained by potential poor hygienic conditions that are conducive to enteric virus transmission as previously reported by Ioulia *et al*. in Greece in 2019, where HPeV was detected in children living in dirty, unhygienic conditions [44]. However, this hypothesis could not be tested in this retrospective study since hygienic conditions were not available in this study. This high HPeV prevalence is much more higher than those found in most parts of the world, such as Asia, Europe, America and Africa [27,28,33,41,45]. The prevalence found in this study is higher than some recent studies conducted worldwide and even in Africa. The prevalence reported in Iran and Greece was 4% (4/100) and 6% (4/71), respectively [45,46]. Our results are slightly higher than those obtained in neighbouring countries such as Niger, where a unique HPeV A-15 isolate has been reported [47]. Nevertheless, some previous studies reported higher HPeV detection prevalence than those described here. Studies conducted in Malawi, Ghana and Egypt showed HPeV prevalences as high as 57% (427/749), 24% (164/682) and 19% (19/100), respectively; confirming that HPeV circulation may be intensive in some African countries [31,32,48]. Variations in the HPeV prevalences between studies could be explained by differences in the socio-demographic and hygienic conditions that may favour or limit enteric virus transmission between specific populations in different epidemiological settings. Another differential factor between the HPeV prevalence we found and that in previous studies could be related to the study populations with different inclusion criteria, such as AFP children versus diarrhoea or gastroenteritis cases. The HPeV prevalence in this study is comparable to the 10.9% reported by Gelaw and colleagues in children with diarrhoea in northern Ethiopia in 2020 [30].

This study showed a significant association between HPeV prevalence and other factors such as age and year of AFP onset. The association between children’s age and the occurrence of HPeV infection of the nervous system was previously described by Olijve and colleagues in 2017. They found that children under 2 years of age are the most at risk of developing symptomatic HPeV infection; those under 6 months of age are specifically more likely to suffer from a severe course of disease, while HPeV-6 mainly affects children over one year of age [7,49]. In this study, it is likely that children < 2 years of age are less susceptible to enteric transmission of HPeV because breastfeeding may have protected them either from virus ingestion or from active viral replication due to passive protection by maternal antibodies. After 2 years, children are more exposed to enteric infection as they begin to eat and drink available food and water.

In this study, males were more affected than females. This observation has been reported in other settings around the world, notably in Denmark where Nielsen and colleagues in 2015 found that, apart from sex, other early environmental risk factors such as mode of delivery, Apgar score, birth weight and gestational age were not associated with HPeV infection (P>0.05) [50,51]. The higher proportion of females in this study may have contributed to the higher prevalence obtained in Cameroon compared to other studies that reported lower HPeV prevalence [35]. Although there is an association between the sex and HPeV prevalence, this factor is only loosely significant because only a few numbers of children with known age were analyzed. The monthly distribution of HPeV detections showed yearly circulation with peaks of detection during rainy seasons. This observation on the seasonality of HPeV circulation has been described by other authors [35,52].

The RT-nested PCR positivity rate of the HPeV-positive samples was only 59% compared to a higher rate of 64.7% reported by Benchop and colleagues in 2008 [32]. One possible reason why some samples were not amplified by nested rRT-PCR is that there may be divergent viruses in some samples that were refractory to the primer sets used. Another reason could be that some of the enzymes used (reverse transcriptase and polymerase) may have had different amplification efficiencies, affecting the VP1-based RT-PCR positivity rate of the viruses initially detected the diagnostic 5’-UTR-based diagnostic RT-PCR; as suggested by Liu and colleagues in 2019 [20]. However, real-time PCR is recognized to be more sensitive than conventional RT-nested PCR.

This study provided newly determined VP1 coding gene sequences for 12 HPeV showing a wide diversity of 1 HPeV type circulating in Cameroon. Phylogenetic analyses assigned all study viruses to the HPeV A and specifically to the specific genotypes HPeV A-1, A-4, A-5, A-10, A-14, A-15, A-17, and A-18. This is consistent with the fact that HPeV is known to have the highest genetic diversity in Africa [30,31,33,34]. However, given that a proportion (9/22) of HPeV-positive samples failed to amplify by nested PCR, as in other studies, it cannot be excluded that a higher number of HPeV genotypes co-circulate in Cameroon, so far without known associated epidemics. Together with a previous report [53], this study suggests that the use of more powerful molecular approaches, such as sensitive and unbiased high-throughput sequencing, would be helpful for an exhaustive assessment of the genetic diversity of co-circulating HPeVs in Cameroon.

Overall, the temporal distribution and phylogenetic characteristics of the studied HPeVs suggest an endemic, rather epidemic, circulation of HPeV in the study population as the studied viruses did not define a monophyletic lineage. Interestingly, the studied HPeV A-1 sequences were very close but distinct to its counterparts (accession numbers MH933780 and MH933781) originating from gastroenteritis patients in Cameroon in 2014 [35]. Other investigated HPeV genotypes A-4, A-5, A-10, A-14, A-15, A-17, and A-18 were reported for the first time in Cameroon. Based on NCBI BLAST search and phylogenetic analyses, their respective closest nucleotide sequence matches (viruses DQ315670, KY931611, JX219568, MG571814, JX219573, KT319121, and KT879915) originated from countries distant from Cameroon: Netherland, Ghana, Bangladesh, Venezuela, Bangladesh, Ivory Coast and China, respectively. This indicates a fundamental gap in the available molecular data on HPeV in Cameroon and Central Africa in general. This study provides substantial data of interest to fill this fundamental gap in the molecular epidemiology of HPeV in Cameroon.

Apart from the 25 AFP cases without known date of disease onset (S1 Table), the time elapsed between AFP onset and stool collection from the remaining 175 AFP cases ranged from 0 to 33 days. Most (92%, 161/175) of the stool samples with known dates were collected within the 14 days of AFP onset whereas the remaining 14 samples were collected more than 14 days after the disease onset. Unfortunately, this study could not rule out the potential association between HePV infection and the AFP observed in the studied patients. A prospective and longitudinal study, including collection of sera and stool samples at different time points would be of great interest to rule out potential association between HPeV detection and AFP in Cameroon.

In contrast to other studies conducted worldwide, such as in Europe and in Iran, where only a few different genotypes are usually described [44,46,54], this study identified 8 different genotypes among the 12 newly sequenced HPeVs. HPeV A-1 and A-5 genotypes, which have long been described mainly in Africa were also detected in this study, thus confirming previous observations [33,45,46]. HPeV A-15 and A-17 genotypes which were rarely reported in Africa, were found in this study [7,43,55]. This suggests their silent circulation in the population. As significant viruses detected in this study did not amplify in RT-nested PCR, further studies, using more powerful sequencing approaches such as next generation sequencing would likely reveal a more diverse genetic landscape of HPeV among AFP patients in Cameroon.

## Conclusion

This study suggests that different HPeV genotypes co-circulate year-round among children with AFP in Cameroon with potential peaks during the rainy season. The study revealed multiple phylogenetic variants segregating into 8 genotypes without a pattern of epidemic circulation. This study also indicates that HPeVs should be considered in the virological investigation of enteric and neurologic disease syndromes in Cameroon and neighboring countries.

## Supporting information

S1 Table Summary of the studied acute flaccid paralysis cases with their sociodemographic features, cell culture and molecular results. (PDF)
