# Supplementary material for: Detection and genetic diversity of parechoviruses in children with acute flaccid paralysis in Cameroon
Source: PLoS One. 2024 May 29;19(5):e0301771. doi: 10.1371/journal.pone.0301771 (PMC11135751; doi:10.1371/journal.pone.0301771)
Supplement: S1 Table — (PDF) [file pone.0301771.s001.pdf]

Supporting information

S1 Table. Summary of the studied Acute Flaccid Paralysis cases with their sociodemographic features, cell culture and molecular results (PDF)

Country Code	Sexe	Ages (years)	Date of AFP Onset	Stool specimen Code	Date of stool collection	Month of stool collection	Interval between AFP Onset and stool collection	Year of stool collection	Final Cell Culture Result on L20B, RD and HE-2c cell lines	Case Or Contact	Region of origin	5'-UTR rRTPCR CT Values	Final VP1 RT-snPCR Results	Assigned HPeV genotype
CAE	M	8	2-Jan-18	CAE-S-001	9-Jan-18	Jan-2018	7	2018	2-Negative	1-Case	Far North	Undetermined	Not aplicable	/
CAE	M	2	4-Jan-18	CAE-S-002	8-Jan-18	Jan-2018	4	2018	2-Negative	1-Case	ADAMAOUA	24.26	Positive	HPeV A18
CAE	M	2	7-Jan-18	CAE-S-003	11-Jan-18	Jan-2018	4	2018	2-Negative	1-Case	Far North	Undetermined	Not aplicable	/
CAE	F	9	13-Feb-18	CAE-S-004	24-Feb-18	Feb-2018	11	2018	2-Negative	1-Case	LITTORAL	Undetermined	Not aplicable	/
CAE	M	11	22-Feb-18	CAE-S-005	4-Mar-18	Mar-18	10	2018	2-Negative	1-Case	LITTORAL	18.22	Positive	HPeV A4
CAE	M	4	3-Mar-18	CAE-S-006	4-Mar-18	Mar-2018	1	2018	2-Negative	1-Case	North	Undetermined	Not aplicable	/
CAE	F	3	27-Feb-18	CAE-S-007	7-Mar-18	Mar-2018	8	2018	2-Negative	1-Case	LITTORAL	Undetermined	Not aplicable	/
CAE	M	Missing	28-Feb-18	CAE-S-008	7-Mar-18	Mar-2018	7	2018	2-Negative	1-Case	North WEast	Undetermined	Not aplicable	/
CAE	M	8	7-Mar-18	CAE-S-009	10-Mar-18	Mar-2018	3	2018	2-Negative	1-Case	North	Undetermined	Not aplicable	/
CAE	M	6	12-Mar-18	CAE-S-010	13-Mar-18	Mar-2018	1	2018	2-Negative	1-Case	Far North	Undetermined	Not aplicable	/
CAE	M	5	11-Mar-18	CAE-S-011	14-Mar-18	Mar-18	3	2018	2-Negative	1-Case	LITTORAL	Undetermined	Not aplicable	/
CAE	M	3	2-Mar-18	CAE-S-012	15-Mar-18	Mar-2018	13	2018	2-Negative	1-Case	ADAMAOUA	Undetermined	Not aplicable	/
CAE	M	1.5	19-Feb-18	CAE-S-013	21-Mar-18	Mar-2018	11	2018	2-Negative	1-Case	Far North	Undetermined	Not aplicable	/

CAE	F	6	16-Mar-18	CAE-S-014	19-Mar-18	Mar-2018	3	2018	2-Negative	1-Case	Far North	Undetermined	Not aplicable	/
CAE	M	5	20-Mar-18	CAE-S-015	24-Mar-18	Mar-2018	4	2018	2-Negative	1-Case	East	Undetermined	Not aplicable	/
CAE	M	4	4-Mar-18	CAE-S-016	25-Mar-18	Mar-2018	21	2018	2-Negative	1-Case	Far North	Undetermined	Not aplicable	/
CAE	M	0.58	18-Mar-18	CAE-S-017	26-Mar-18	Mar-2018	8	2018	2-Negative	1-Case	Far North	Undetermined	Not aplicable	/
CAE	F	4	22-Mar-18	CAE-S-018	27-Mar-18	Mar-2018	5	2018	2-Negative	1-Case	ADAMAOUA	Undetermined	Not aplicable	/
CAE	M	13	24-Mar-18	CAE-S-019	29-Mar-18	Mar-18	5	2018	2-Negative	1-Case	ADAMAOUA	27.11	Negative	/
CAE	M	2.33	29-Mar-18	CAE-S-020	31-Mar-18	Mar-2018	2	2018	2-Negative	1-Case	East	Undetermined	Not aplicable	/
CAE	M	6.16	25-Mar-18	CAE-S-021	29-Mar-18	Mar-2018	4	2018	2-Negative	1-Case	Far North	20.25357603	Negative	/
CAE	F	2.3	27-Mar-18	CAE-S-022	3-Apr-18	Apr-2018	7	2018	2-Negative	1-Case	CENTRE	Undetermined	Not aplicable	/
CAE	M	4	1-Apr-18	CAE-S-023	2-Apr-18	Apr-2018	1	2018	2-Negative	1-Case	LITTORAL	Undetermined	Not aplicable	/
CAE	M	4	2-Apr-18	CAE-S-024	4-Apr-18	Apr-2018	2	2018	2-Negative	1-Case	Far North	Undetermined	Not aplicable	/
CAE	F	Missing	31-Mar-18	CAE-S-025	8-Apr-18	Apr-18	8	2018	2-Negative	1-Case	North	Undetermined	Not aplicable	/
CAE	M	8	10-Apr-18	CAE-S-026	11-Apr-18	Apr-2018	1	2018	2-Negative	1-Case	CENTRE	Undetermined	Not aplicable	/
CAE	M	5	8-Apr-18	CAE-S-027	10-Apr-18	Apr-2018	2	2018	2-Negative	1-Case	North	Undetermined	Not aplicable	/
CAE	M	9	3-Apr-18	CAE-S-028	13-Apr-18	Apr-2018	10	2018	2-Negative	1-Case	ADAMAOUA	Undetermined	Not aplicable	/
CAE	F	11	17-Apr-18	CAE-S-029	18-Apr-18	Apr-18	1	2018	2-Negative	1-Case	CENTRE	Undetermined	Not aplicable	/
CAE	F	2	13-Apr-18	CAE-S-030	16-Apr-18	Apr-2018	3	2018	2-Negative	1-Case	South WEast	Undetermined	Not aplicable	/
CAE	M	4	6-Apr-18	CAE-S-031	15-Apr-18	Apr-2018	9	2018	2-Negative	1-Case	ADAMAOUA	Undetermined	Not aplicable	/
CAE	M	Missing	10-Apr-18	CAE-S-032	16-Apr-18	Apr-2018	6	2018	2-Negative	1-Case	WEast	Undetermined	Not aplicable	/

CAE	M	Missing	15-Apr-18	CAE-S-033	17-Apr-18	Apr-2018	2	2018	2-Negative	1-Case	North WEast	Undetermined	Not aplicable	/
CAE	F	2.5	16-Apr-18	CAE-S-034	19-Apr-18	Apr-2018	3	2018	2-Negative	1-Case	East	Undetermined	Not aplicable	/
CAE	M	3	18-Apr-18	CAE-S-035	23-Apr-18	Apr-18	5	2018	2-Negative	1-Case	LITTORAL	Undetermined	Not aplicable	/
CAE	F	1.25	20-Apr-18	CAE-S-036	26-Apr-18	Apr-2018	6	2018	2-Negative	1-Case	CENTRE	Undetermined	Not aplicable	/
CAE	M	Missing	8-Apr-18	CAE-S-037	29-Apr-18	Apr-2018	21	2018	2-Negative	1-Case	Far North	Undetermined	Not aplicable	/
CAE	F	Missing	28-Apr-18	CAE-S-038	30-Apr-18	Apr-2018	2	2018	2-Negative	1-Case	WEast	Undetermined	Not aplicable	/
CAE	F	11	22-Apr-18	CAE-S-039	30-Apr-18	Apr-18	8	2018	2-Negative	1-Case	North	Undetermined	Not aplicable	/
CAE	F	13	25-Apr-18	CAE-S-040	2-May-18	May-2018	7	2018	2-Negative	1-Case	ADAMAOUA	Undetermined	Not aplicable	/
CAE	F	Missing	2-May-18	CAE-S-041	7-May-18	May-2018	5	2018	2-Negative	1-Case	North WEast	Undetermined	Not aplicable	/
CAE	M	Missing	5-May-18	CAE-S-042	9-May-18	May-2018	4	2018	2-Negative	1-Case	WEast	Undetermined	Not aplicable	/
CAE	M	4	7-May-18	CAE-S-043	8-May-18	May-18	1	2018	2-Negative	1-Case	LITTORAL	Undetermined	Not aplicable	/
CAE	M	1.58	9-May-18	CAE-S-044	12-May-18	May-2018	3	2018	2-Negative	1-Case	LITTORAL	Undetermined	Not aplicable	/
CAE	F	1.41	11-May-18	CAE-S-045	12-May-18	May-2018	1	2018	2-Negative	1-Case	LITTORAL	Undetermined	Not aplicable	/
CAE	F	1.16	15-May-18	CAE-S-046	15-May-18	May-2018	0	2018	2-Negative	1-Case	East	Undetermined	Not aplicable	/
CAE	M	9.6	23-May-18	CAE-S-047	28-May-18	May-2018	5	2018	2-Negative	1-Case	CENTRE	Undetermined	Not aplicable	/
CAE	M	4.16	31-May-18	CAE-S-048	1-Jun-18	Jun-2018	1	2018	2-Negative	1-Case	CENTRE	Undetermined	Not aplicable	/
CAE	M	7	29-May-18	CAE-S-049	4-Jun-18	Jun-2018	6	2018	2-Negative	1-Case	East	Undetermined	Not aplicable	/
CAE	F	1	1-Jun-18	CAE-S-050	5-Jun-18	Jun-2018	4	2018	2-Negative	1-Case	ADAMAOUA	Undetermined	Not aplicable	/

CAE	F	9.75	8-Jun-18	CAE-S-051	11-Jun-18	Jun-2018	3	2018	2-Negative	1-Case	ADAMAOUA	Undetermined	Not aplicable	/
CAE	M	4	10-Jun-18	CAE-S-052	12-Jun-18	Jun-18	2	2018	2-Negative	1-Case	ADAMAOUA	Undetermined	Not aplicable	/
CAE	M	2	22-Jun-18	CAE-S-053	4-Jul-18	Jul-2018	12	2018	2-Negative	1-Case	East	Undetermined	Not aplicable	/
CAE	F	1.58	11-Jun-18	CAE-S-054	26-Jun-18	Jun-2018	15	2018	2-Negative	1-Case	Far North	Undetermined	Not aplicable	/
CAE	F	2.5	1-Jul-18	CAE-S-055	7-Jul-18	Jul-2018	6	2018	2-Negative	1-Case	LITTORAL	Undetermined	Not aplicable	/
CAE	F	8	8-Jul-18	CAE-S-056	13-Jul-18	Jul-2018	5	2018	2-Negative	1-Case	Far North	Undetermined	Not aplicable	/
CAE	M	1.83	8-Jul-18	CAE-S-057	14-Jul-18	Jul-2018	6	2018	2-Negative	1-Case	ADAMAOUA	27.06	Negative	/
CAE	F	Missing	12-Jul-18	CAE-S-058	23-Jul-18	Jul-18	11	2018	2-Negative	1-Case	North WEast	Undetermined	Not aplicable	/
CAE	F	8.58	9-Jul-18	CAE-S-059	19-Jul-18	Jul-2018	10	2018	2-Negative	1-Case	Far North	Undetermined	Not aplicable	/
CAE	M	4	27-Jul-18	CAE-S-060	28-Jul-18	Jul-2018	1	2018	2-Negative	1-Case	CENTRE	Undetermined	Not aplicable	/
CAE	M	Missing	15-Jul-18	CAE-S-061	28-Jul-18	Jul-2018	13	2018	2-Negative	1-Case	Far North	Undetermined	Not aplicable	/
CAE	M	1.3	1-Aug-18	CAE-S-062	5-Aug-18	Aug-2018	4	2018	2-Negative	1-Case	CENTRE	Undetermined	Not aplicable	/
CAE	M	2	27-Jul-18	CAE-S-063	8-Aug-18	Aug-2018	12	2018	2-Negative	1-Case	South	Undetermined	Not aplicable	/
CAE	F	3	7-Aug-18	CAE-S-064	12-Aug-18	Aug-2018	5	2018	2-Negative	1-Case	LITTORAL	Undetermined	Not aplicable	/
CAE	M	12	6-Aug-18	CAE-S-065	13-Aug-18	Aug-2018	7	2018	2-Negative	1-Case	LITTORAL	Undetermined	Not aplicable	/
CAE	M	Missing	10-Aug-18	CAE-S-066	13-Aug-18	Aug-18	3	2018	2-Negative	1-Case	North	Undetermined	Not aplicable	/
CAE	M	1.3	20-Aug-18	CAE-S-067	22-Aug-18	Aug-2018	2	2018	2-Negative	1-Case	ADAMAOUA	Undetermined	Not aplicable	/
CAE	F	1.5	12-Aug-18	CAE-S-068	25-Aug-18	Aug-2018	13	2018	2-Negative	1-Case	ADAMAOUA	Undetermined	Not aplicable	/

CAE	M	8	18-Aug-18	CAE-S-069	23-Aug-18	Aug-2018	5	2018	2-Negative	1-Case	North	Undetermined	Not aplicable	/
CAE	M	Missing	24-Aug-18	CAE-S-070	27-Aug-18	Aug-2018	3	2018	2-Negative	1-Case	WEast	Undetermined	Not aplicable	/
CAE	M	3	1-Sep-18	CAE-S-071	4-Sep-18	Sep-2018	3	2018	2-Negative	1-Case	North	Undetermined	Not aplicable	/
CAE	F	Missing	30-Aug-18	CAE-S-072	6-Sep-18	Sep-18	7	2018	2-Negative	1-Case	WEast	Undetermined	Not aplicable	/
CAE	M	3.91	27-Aug-18	CAE-S-073	7-Sep-18	Sep-2018	11	2018	2-Negative	1-Case	Far North	Undetermined	Not aplicable	/
CAE	M	7	7-Sep-18	CAE-S-074	9-Sep-18	Sep-2018	2	2018	2-Negative	1-Case	Far North	Undetermined	Not aplicable	/
CAE	F	Missing	5-Sep-18	CAE-S-075	14-Sep-18	Sep-2018	9	2018	2-Negative	1-Case	Far North	Undetermined	Not aplicable	/
CAE	F	4	14-Sep-18	CAE-S-076	21-Sep-18	Sep-18	7	2018	2-Negative	1-Case	North	Undetermined	Not aplicable	/
CAE	M	3	16-Sep-18	CAE-S-077	25-Sep-18	Sep-2018	9	2018	2-Negative	1-Case	Far North	26.02	Negative	/
CAE	F	Missing	26-Sep-18	CAE-S-078	30-Sep-18	Sep-2018	4	2018	2-Negative	1-Case	WEast	Undetermined	Not aplicable	/
CAE	M	Missing	29-Sep-18	CAE-S-079	1-Oct-18	Oct-2018	2	2018	2-Negative	1-Case	WEast	Undetermined	Not aplicable	/
CAE	M	1.91	8-Oct-18	CAE-S-080	10-Oct-18	Oct-2018	2	2018	2-Negative	1-Case	East	Undetermined	Not aplicable	/
CAE	M	6	9-Oct-18	CAE-S-081	15-Oct-18	Oct-2018	6	2018	2-Negative	1-Case	South	Undetermined	Not aplicable	/
CAE	M	Missing	23-Sep-18	CAE-S-082	14-Oct-18	Oct-2018	21	2018	2-Negative	1-Case	WEast	Undetermined	Not aplicable	/
CAE	M	Missing	17-Oct-18	CAE-S-083	18-Oct-18	Oct-2018	1	2018	2-Negative	1-Case	WEast	Undetermined	Not aplicable	/
CAE	M	7	7-Oct-18	CAE-S-084	18-Oct-18	Oct-18	11	2018	2-Negative	1-Case	East	27.98	Negative	/
CAE	M	1.91	8-Oct-18	CAE-S-085	20-Oct-18	Oct-2018	12	2018	2-Negative	1-Case	Far North	Undetermined	Not aplicable	/
CAE	M	7	21-Oct-18	CAE-S-086	25-Oct-18	Oct-2018	4	2018	2-Negative	1-Case	CENTRE	Undetermined	Not aplicable	/
CAE	M	Missing	22-Oct-18	CAE-S-087	29-Oct-18	Oct-2018	7	2018	2-Negative	1-Case	WEast	Undetermined	Not aplicable	/

CAE	M	Missing	25-Oct-18	CAE-S-088	31-Oct-18	Oct-2018	6	2018	2-Negative	1-Case	North WEast	Undetermined	Not aplicable	/
CAE	M	11	19-Oct-18	CAE-S-089	30-Oct-18	Oct-2018	11	2018	2-Negative	1-Case	Far North	Undetermined	Not aplicable	/
CAE	F	4.16	4-Nov-18	CAE-S-090	4-Nov-18	Nov-18	0	2018	2-Negative	1-Case	North	Undetermined	Not aplicable	/
CAE	M	Missing	5-Nov-18	CAE-S-091	8-Nov-18	Nov-2018	3	2018	2-Negative	1-Case	WEast	Undetermined	Not aplicable	/
CAE	F	4	4-Nov-18	CAE-S-092	7-Nov-18	Nov-2018	3	2018	2-Negative	1-Case	Far North	Undetermined	Not aplicable	/
CAE	F	2.08	5-Nov-18	CAE-S-093	7-Nov-18	Nov-2018	2	2018	2-Negative	1-Case	Far North	Undetermined	Not aplicable	/
CAE	M	8.75	2-Nov-18	CAE-S-094	7-Nov-18	Nov-2018	5	2018	2-Negative	1-Case	Far North	22.46734887	Negative	/
CAE	F	5	Unknown	CAE-S-095	8-Nov-18	Nov-2018	/	2018	2-Negative	1-Case	Far North	Undetermined	Not aplicable	/
CAE	M	3	7-Nov-18	CAE-S-096	12-Nov-18	Nov-2018	5	2018	2-Negative	1-Case	CENTRE	Undetermined	Not aplicable	/
CAE	M	0	5-Nov-18	CAE-S-097	10-Nov-18	Nov-2018	5	2018	2-Negative	1-Case	North	18.56	Positive	HPeV A15
CAE	F	3.08	23-Oct-18	CAE-S-098	12-Nov-18	Nov-18	20	2018	2-Negative	1-Case	WEast	Undetermined	Not aplicable	/
CAE	M	2	11-Nov-18	CAE-S-099	12-Nov-18	Nov-2018	1	2018	2-Negative	1-Case	CENTRE	Undetermined	Not aplicable	/
CAE	M	7	6-Nov-18	CAE-S-100	13-Nov-18	Nov-2018	7	2018	2-Negative	1-Case	Far North	Undetermined	Not aplicable	/
CAE	F	11	11-Nov-18	CAE-S-101	13-Nov-18	Nov-2018	2	2018	2-Negative	1-Case	LITTORAL	Undetermined	Not aplicable	/
CAE	F	6	10-Nov-18	CAE-S-102	14-Nov-18	Nov-2018	4	2018	2-Negative	1-Case	South WEast	24.90863649	Positive	HPeV A1
CAE	M	2.66	Unknown	CAE-S-103	15-Nov-18	Nov-2018	/	2018	2-Negative	1-Case	CENTRE	Undetermined	Not aplicable	/
CAE	F	Missing	18-Nov-18	CAE-S-104	18-Nov-18	Nov-18	0	2018	2-Negative	1-Case	WEast	Undetermined	Not aplicable	/
CAE	M	4.5	16-Nov-18	CAE-S-105	21-Nov-18	Nov-2018	5	2018	2-Negative	1-Case	North	Undetermined	Not aplicable	/
CAE	M	Missing	19-Nov-18	CAE-S-106	20-Nov-18	Nov-2018	1	2018	2-Negative	1-Case	WEast	Undetermined	Not aplicable	/

CAE	F	5	28-Nov-18	CAE-S-107	4-Dec-18	Dec-2018	6	2018	2-Negative	1-Case	Far North	Undetermined	Not aplicable	/
CAE	M	2.16	30-Oct-18	CAE-S-108	11-Dec-18	Dec-18	11	2018	2-Negative	1-Case	Far North	24.58	Positive	HPeV A10
CAE	M	4	11-Dec-18	CAE-S-109	26-Dec-18	Dec-2018	15	2018	2-Negative	1-Case	Far North	26.41	Positive	HPeV A14
CAE	F	Missing	Unknown	CAE-S-110	4-Jan-19	Jan-2019	/	2019	2-Negative	1-Case	North	Undetermined	Not aplicable	/
CAE	F	5	3-Jan-19	CAE-S-111	5-Jan-19	Jan-2019	2	2019	2-Negative	1-Case	CENTRE	Undetermined	Not aplicable	/
CAE	M	Missing	16-Jan-19	CAE-S-112	20-Jan-19	Jan-2019	4	2019	2-Negative	1-Case	WEast	Undetermined	Not aplicable	/
CAE	M	0	20-Jan-19	CAE-S-113	26-Jan-19	Jan-2019	6	2019	2-Negative	1-Case	North	Undetermined	Not aplicable	/
CAE	M	2.58	21-Jan-19	CAE-S-114	27-Jan-19	Jan-2019	6	2019	2-Negative	1-Case	East	Undetermined	Not aplicable	/
CAE	F	4	13-Jan-19	CAE-S-115	30-Jan-19	Jan-19	17	2019	2-Negative	1-Case	LITTORAL	Undetermined	Not aplicable	/
CAE	F	Missing	24-Jan-19	CAE-S-116	30-Jan-19	Jan-2019	6	2019	2-Negative	1-Case	WEast	Undetermined	Not aplicable	/
CAE	F	6	16-Feb-19	CAE-S-117	19-Feb-19	Feb-2019	3	2019	2-Negative	1-Case	CENTRE	Undetermined	Not aplicable	/
CAE	F	14.41	7-Feb-19	CAE-S-118	19-Feb-19	Feb-2019	12	2019	2-Negative	1-Case	CENTRE	Undetermined	Not aplicable	/
CAE	F	Missing	12-Feb-19	CAE-S-119	23-Feb-19	Feb-2019	11	2019	2-Negative	1-Case	WEast	Undetermined	Not aplicable	/
CAE	F	Missing	7-Mar-19	CAE-S-120	8-Mar-19	Mar-2019	1	2019	2-Negative	1-Case	WEast	Undetermined	Not aplicable	/
CAE	M	Missing	Unknown	CAE-S-121	17-Mar-19	Mar-19	/	2019	2-Negative	1-Case	North	Undetermined	Not aplicable	/
CAE	M	Missing	11-Mar-19	CAE-S-122	19-Mar-19	Mar-2019	8	2019	2-Negative	1-Case	WEast	Undetermined	Not aplicable	/
CAE	M	5	18-Mar-19	CAE-S-123	26-Mar-19	Mar-2019	8	2019	2-Negative	1-Case	South	Undetermined	Not aplicable	/
CAE	F	9	19-Mar-19	CAE-S-124	27-Mar-19	Mar-2019	8	2019	2-Negative	1-Case	South	Undetermined	Not aplicable	/
CAE	F	4.25	8-Mar-19	CAE-S-125	29-Mar-19	Mar-2019	21	2019	2-Negative	1-Case	North	Undetermined	Not aplicable	/

CAE	F	Missing	21-Mar-19	CAE-S-126	31-Mar-19	Mar-2019	10	2019	2-Negative	1-Case	South	26.64	Positive	HPeV A1
CAE	M	Missing	Unknown	CAE-S-127	31-Mar-19	Mar-2019	/	2019	2-Negative	1-Case	Far North	27.81466991	Positive	HPeV A17
CAE	F	13	27-Mar-19	CAE-S-128	30-Mar-19	Mar-2019	3	2019	2-Negative	1-Case	North	Undetermined	Not aplicable	/
CAE	F	8.33	27-Mar-19	CAE-S-129	31-Mar-19	Mar-19	4	2019	2-Negative	1-Case	North	Undetermined	Not aplicable	/
CAE	M	1.83	Unknown	CAE-S-130	30-Mar-19	Mar-2019	/	2019	2-Negative	1-Case	North	Undetermined	Not aplicable	/
CAE	M	Missing	29-Mar-19	CAE-S-131	1-Apr-19	Apr-2019	3	2019	2-Negative	1-Case	North WEast	Undetermined	Not aplicable	/
CAE	F	11.16	1-Apr-19	CAE-S-132	2-Apr-19	Apr-2019	1	2019	2-Negative	1-Case	CENTRE	Undetermined	Not aplicable	/
CAE	M	4.41	7-Apr-19	CAE-S-133	11-Apr-19	Apr-2019	4	2019	2-Negative	1-Case	CENTRE	Undetermined	Not aplicable	/
CAE	M	4	2-Apr-19	CAE-S-134	9-Apr-19	Apr-2019	7	2019	2-Negative	1-Case	East	Undetermined	Not aplicable	/
CAE	M	3	3-Apr-19	CAE-S-135	12-Apr-19	Apr-19	9	2019	2-Negative	1-Case	CENTRE	Undetermined	Not aplicable	/
CAE	F	1	6-Mar-19	CAE-S-136	14-Apr-19	Apr-2019	33	2019	2-Negative	1-Case	Far North	Undetermined	Not aplicable	/
CAE	M	3	Unknown	CAE-S-137	14-Apr-19	Apr-2019	/	2019	2-Negative	1-Case	Far North	Undetermined	Not aplicable	/
CAE	F	2	21-Apr-19	CAE-S-138	25-Apr-19	Apr-2019	/	2019	2-Negative	1-Case	CENTRE	Undetermined	Not aplicable	/
CAE	F	6	Unknown	CAE-S-139	29-Apr-19	Apr-19	/	2019	2-Negative	1-Case	North	Undetermined	Not aplicable	/
CAE	M	2.3	27-Apr-19	CAE-S-140	30-Apr-19	Apr-2019	3	2019	2-Negative	1-Case	ADAMAOUA	Undetermined	Not aplicable	/
CAE	M	Missing	Unknown	CAE-S-141	1-May-19	May-2019	/	2019	2-Negative	1-Case	ADAMAOUA	Undetermined	Not aplicable	/
CAE	M	Missing	Unknown	CAE-S-142	4-May-19	May-2019	/	2019	2-Negative	1-Case	North	23.24	Positive	HPeV A14
CAE	M	6	29-May-19	CAE-S-143	6-Jun-19	Jun-2019	8	2019	2-Negative	1-Case	North	Undetermined	Not aplicable	/
CAE	M	2	Unknown	CAE-S-144	20-Jun-19	Jun-2019	/	2019	2-Negative	1-Case	South WEast	Undetermined	Not aplicable	/

CAE	F	Missing	12-Jun-19	CAE-S-145	19-Jun-19	Jun-19	7	2019	2-Negative	1-Case	WEast	Undetermined	Not aplicable	/
CAE	M	Missing	15-Jun-19	CAE-S-146	22-Jun-19	Jun-2019	7	2019	2-Negative	1-Case	WEast	Undetermined	Not aplicable	/
CAE	M	1.5	Unknown	CAE-S-147	13-Jul-19	Jul-2019	/	2019	2-Negative	1-Case	Far North	26.05681938	Positive	unusable sequence
CAE	F	4.16	15-Jul-19	CAE-S-148	22-Jul-19	Jul-2019	/	2019	2-Negative	1-Case	ADAMAOUA	Undetermined	Not aplicable	/
CAE	M	Missing	Unknown	CAE-S-149	23-Jul-19	Jul-2019	/	2019	2-Negative	1-Case	North WEast	Undetermined	Not aplicable	/
CAE	F	2	Unknown	CAE-S-150	28-Jul-19	Jul-2019	/	2019	2-Negative	1-Case	ADAMAOUA	Undetermined	Not aplicable	/
CAE	F	Missing	27-Jul-19	CAE-S-151	30-Jul-19	Jul-19	3	2019	2-Negative	1-Case	South	Undetermined	Not aplicable	/
CAE	M	1.58	14-Aug-19	CAE-S-152	21-Aug-19	Aug-2019	7	2019	2-Negative	1-Case	LITTORAL	Undetermined	Not aplicable	/
CAE	F	Missing	18-Aug-19	CAE-S-153	20-Aug-19	Aug-2019	2	2019	2-Negative	1-Case	North WEast	Undetermined	Not aplicable	/
CAE	M	4	12-Aug-19	CAE-S-154	26-Aug-19	Aug-2019	14	2019	2-Negative	1-Case	LITTORAL	Undetermined	Not aplicable	/
CAE	M	4	Unknown	CAE-S-155	4-Sep-19	Sep-2019	/	2019	2-Negative	1-Case	East	Undetermined	Not aplicable	/
CAE	M	Missing	22-Aug-19	CAE-S-156	26-Aug-19	Aug-2019	4	2019	2-Negative	1-Case	North WEast	Undetermined	Not aplicable	/
CAE	M	Missing	8-Aug-19	CAE-S-157	7-Sep-19	Sep-2019	30	2019	2-Negative	1-Case	South	Undetermined	Not aplicable	/
CAE	M	Missing	Unknown	CAE-S-158	7-Sep-19	Sep-2019	/	2019	2-Negative	1-Case	South	Undetermined	Not aplicable	/
CAE	F	1.16	19-Sep-19	CAE-S-159	23-Sep-19	Sep-19	4	2019	2-Negative	1-Case	CENTRE	Undetermined	Not aplicable	/
CAE	M	7	18-Sep-19	CAE-S-160	26-Sep-19	Sep-2019	8	2019	2-Negative	1-Case	South	25.20	Positive	HPeV A5
CAE	M	1.58	28-Sep-19	CAE-S-161	28-Sep-19	Sep-2019	0	2019	2-Negative	1-Case	East	Undetermined	Not aplicable	/
CAE	M	5	26-Sep-19	CAE-S-162	28-Sep-19	Sep-2019	0	2019	2-Negative	1-Case	LITTORAL	Undetermined	Not aplicable	/

CAE	M	Missing	17-Sep-19	CAE-S-163	29-Sep-19	Sep-2019	12	2019	2-Negative	1-Case	North	Undetermined	Not aplicable	/
CAE	M	3	27-Sep-19	CAE-S-164	3-Oct-19	Oct-2019	6	2019	2-Negative	1-Case	CENTRE	Undetermined	Not aplicable	/
CAE	M	Missing	Unknown	CAE-S-165	9-Oct-19	Oct-19	/	2019	2-Negative	1-Case	ADAMAOUA	Undetermined	Not aplicable	/
CAE	M	1.58	12-Oct-19	CAE-S-166	13-Oct-19	Oct-2019	1	2019	2-Negative	1-Case	East	Undetermined	Not aplicable	/
CAE	M	3	13-Oct-19	CAE-S-167	14-Oct-19	Oct-2019	1	2019	2-Negative	1-Case	East	Undetermined	Not aplicable	/
CAE	F	Missing	6-Oct-19	CAE-S-168	12-Oct-19	Oct-2019	6	2019	2-Negative	1-Case	WEast	Undetermined	Not aplicable	/
CAE	M	4	7-Oct-19	CAE-S-169	18-Oct-19	Oct-19	11	2019	2-Negative	1-Case	LITTORAL	Undetermined	Not aplicable	/
CAE	F	6	25-Aug-19	CAE-S-170	16-Oct-19	Oct-2019	22	2019	2-Negative	1-Case	East	Undetermined	Not aplicable	/
CAE	M	2.58	Unknown	CAE-S-171	16-Oct-19	Oct-2019	/	2019	2-Negative	1-Case	East	Undetermined	Not aplicable	/
CAE	M	Missing	14-Oct-19	CAE-S-172	22-Oct-19	Oct-2019	8	2019	2-Negative	1-Case	WEast	Undetermined	Not aplicable	/
CAE	F	Missing	19-Oct-19	CAE-S-173	24-Oct-19	Oct-2019	5	2019	2-Negative	1-Case	LITTORAL	Undetermined	Not aplicable	/
CAE	F	4	21-Oct-19	CAE-S-174	28-Oct-19	Oct-2019	7	2019	2-Negative	1-Case	South	20.51	Positive	HPeV A17
CAE	F	6	Unknown	CAE-S-175	30-Oct-19	Oct-19	/	2019	2-Negative	1-Case	CENTRE	Undetermined	Not aplicable	/
CAE	M	0.75	Unknown	CAE-S-176	30-Oct-19	Oct-2019	/	2019	2-Negative	1-Case	Far North	Undetermined	Not aplicable	/
CAE	M	7	Unknown	CAE-S-177	2-Nov-19	Nov-2019	/	2019	2-Negative	1-Case	Far North	Undetermined	Not aplicable	/
CAE	M	5	20-Oct-19	CAE-S-178	31-Oct-19	Oct-2019	11	2019	2-Negative	1-Case	South	Undetermined	Not aplicable	/
CAE	M	2.5	31-Oct-19	CAE-S-179	7-Nov-19	Nov-19	7	2019	2-Negative	1-Case	East	26.78	Negative	/
CAE	F	Missing	20-Oct-19	CAE-S-180	8-Nov-19	Nov-2019	19	2019	2-Negative	1-Case	South	Undetermined	Not aplicable	/
CAE	F	Missing	30-Oct-19	CAE-S-181	13-Nov-19	Nov-2019	14	2019	2-Negative	1-Case	South	Undetermined	Not aplicable	/

CAE	F	Missing	2-Nov-19	CAE-S-182	13-Nov-19	Nov-2019	11	2019	2-Negative	1-Case	South	Undetermined	Not aplicable	/
CAE	M	Missing	1-Nov-19	CAE-S-183	7-Nov-19	Nov-2019	6	2019	2-Negative	1-Case	WEast	Undetermined	Not aplicable	/
CAE	F	4	Unknown	CAE-S-184	17-Nov-19	Nov-2019	/	2019	2-Negative	1-Case	ADAMAOUA	Undetermined	Not aplicable	/
CAE	F	4	16-Nov-19	CAE-S-185	19-Nov-19	Nov-2019	3	2019	2-Negative	1-Case	LITTORAL	Undetermined	Not aplicable	/
CAE	F	Missing	9-Nov-19	CAE-S-186	19-Nov-19	Nov-19	10	2019	2-Negative	1-Case	South	Undetermined	Not aplicable	/
CAE	M	9	26-Oct-19	CAE-S-187	21-Nov-19	Nov-2019	26	2019	2-Negative	1-Case	South WEast	Undetermined	Not aplicable	/
CAE	M	4.33	16-Nov-19	CAE-S-188	25-Nov-19	Nov-2019	9	2019	2-Negative	1-Case	East	27.94238344	Negative	/
CAE	M	3.33	19-Nov-19	CAE-S-189	22-Nov-19	Nov-2019	3	2019	2-Negative	1-Case	Far North	22.50	Positive	HPeV A15
CAE	M	3	Unknown	CAE-S-190	24-Nov-19	Nov-2019	/	2019	2-Negative	1-Case	Far North	Undetermined	Not aplicable	/
CAE	F	4	23-Nov-19	CAE-S-191	27-Nov-19	Nov-2019	4	2019	2-Negative	1-Case	East	Undetermined	Not aplicable	/
CAE	F	5	Unknown	CAE-S-192	30-Nov-19	Nov-2019	/	2019	2-Negative	1-Case	ADAMAOUA	Undetermined	Not aplicable	/
CAE	M	12	21-Nov-19	CAE-S-193	2-Dec-19	Dec-2019	11	2019	2-Negative	1-Case	East	Undetermined	Not aplicable	/
CAE	M	1.41	1-Dec-19	CAE-S-194	3-Dec-19	Dec-2019	2	2019	2-Negative	1-Case	East	Undetermined	Not aplicable	/
CAE	M	4	2-Dec-19	CAE-S-195	7-Dec-19	Dec-2019	5	2019	2-Negative	1-Case	ADAMAOUA	Undetermined	Not aplicable	/
CAE	M	Missing	23-Nov-19	CAE-S-196	10-Dec-19	Dec-19	17	2019	2-Negative	1-Case	South	25.30318439	Negative	/
CAE	M	5	3-Dec-19	CAE-S-197	14-Dec-19	Dec-2019	11	2019	2-Negative	1-Case	South	Undetermined	Not aplicable	/
CAE	M	2.5	16-Dec-19	CAE-S-198	18-Dec-19	Dec-2019	2	2019	2-Negative	1-Case	Far North	Undetermined	Not aplicable	/
CAE	F	1.25	15-Dec-19	CAE-S-199	19-Dec-19	Dec-2019	4	2019	2-Negative	1-Case	ADAMAOUA	Undetermined	Not aplicable	/
CAE	M	5	Unknown	CAE-S-200	24-Dec-19	Dec-19	/	2019	2-Negative	1-Case	Far North	Undetermined	Not aplicable	/
